# Welcome to volume 5 of *Future Science OA*

**DOI:** 10.4155/fsoa-2018-0100

**Published:** 2018-11-23

**Authors:** Francesca Lake

**Affiliations:** 1 Future Science Group, Unitec House, 2 Albert Place, London N3 1QB

**Keywords:** biomedicine, biotechnology, health, medicine, open access, research

Welcome to the first issue of volume 5 from *Future Science OA*! We are delighted to be celebrating our fifth birthday this year, which will also see us surpass 400 publications. In this Foreword, I will take a look back over our highlights from 2018, and look forward to what we have planned in 2019.

2018 has seen us continue to disseminate our content in the traditional ways, as well as the novel. We now see 6% of our readers come through social media, which is a threefold increase on 2017. Furthermore, 2018 saw us partner with ScienceOpen, the research and publishing network. All *Future Science OA* content is now available in a ScienceOpen collection [1]. Here, you can find all of our articles and view some of their metrics. One of our favorite parts of ScienceOpen is the ability to view the reference list of each article in detail, meaning it is easy to find the most relevant, related content to continue your research.

We also supported the second iteration of the Future Science Early Career Research Award [2]. This year saw us receive 19 nominations, from which a shortlist of five finalists was selected by our expert judging panel. The panel this year comprised members of our Editorial Board and Young Ambassador panel, including Joe Abisambra, the winner of the 2017 award. Viviana Mucci was then announced as the winner following a public vote, which saw over 4000 voters choose their winner [3]. We recommend reading her profile as she is doing some fabulous work.

With gender equality in the sciences continuing to be an important topic of discussion, we were delighted to see that all five finalists were outstanding female early career researchers – this bodes well for the future.

We have also been supporting researchers even earlier in their careers this year. The ChrisXandDrake Science Award is an initiative that promotes science in primary schools [4]. This year, the initiative and a team of researcher mentors took 9- to 10-year-old school children through the scientific investigation process, from hypothesis-building through study design to data collection, write-up and publication. We were delighted to attend their poster presentation event, and have since published their write-ups in *Future Science OA* [5]. It was wonderful to see children learning more about the research process, and a delight to hear them talking about their future plans for careers in scientific research.

## Content highlights

This year we have been reaching over 20,000 full-text readers per month, an increase of around 80% on 2017. With article numbers consistent, this highlights that our articles are reaching more and more readers, an indicator of impact and quality. In fact, each of our articles now reaches over 1000 readers on average. This includes readers both directly on our website, and on article versions hosted on PubMed Central.

**Figure 1.**
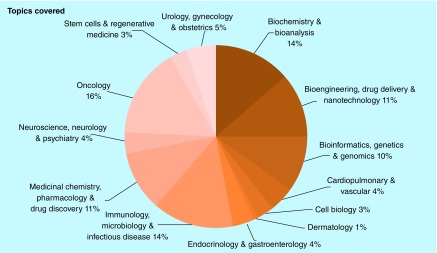
**Topics covered in *Future Science OA* by percentage.**

With the launch of our topic collections in 2017, we are now able to delve further into the overarching topics covered by our articles (Figure 1).

These numbers are perhaps not surprising, given the large amount of research dedicated to the continually ‘hot’ topics that are oncology, drug development and infectious diseases. Our content highlights for the year reflect these hot topics.

Our most-read article for 2018 is an Editorial from Benjamin Satterfield (Mayo Clinic, MN, USA), discussing Nipah virus, a concerning paramyxovirus that is fairly well understood, but for which methods for prevention and cure are lacking [6]. With nearly 3000 downloads, our readers clearly agree that more effort needs to be expended in this field.

Another article on infectious diseases featured in a top spot – a review of antimicrobial resistance mechanisms and novel ways to approach development of new therapeutics [7].

There has also been an increasing interest in recent years in plants as a basis for pharmacological research. Another highlight for the year was a review examining the potential held in rosemary [8].

In terms of research articles, which have made up 75% of our content this year, our highlights include an investigation of whether dietary glutamate could contribute to obsessive–compulsive disorder [9] and an investigation comparing whether what is discussed in social media about oncology research and foods is related to what is published in the scientific literature [10].

## Looking further into journal statistics

We continue to place a high emphasis on author experience and can report that the average time from initial submission to post-peer review decision for 2018 was 36 days. From submission of a revision to final acceptance took an average of 7 days. 100% of our articles are promoted through our social media accounts, and on average we respond to all queries within 2 working days of receipt.

Each year, we also look back over how our readers and authors have changed, to ensure we are reaching a global audience, and still meeting the needs of the worldwide community of authors.

**Figure 2.**
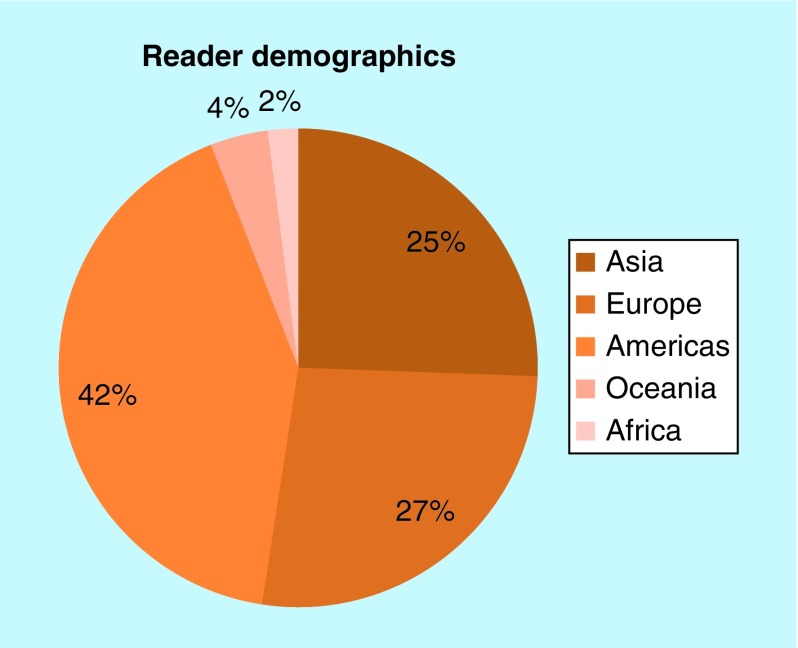
***Future Science OA* reader demographics.**

**Figure 3.**
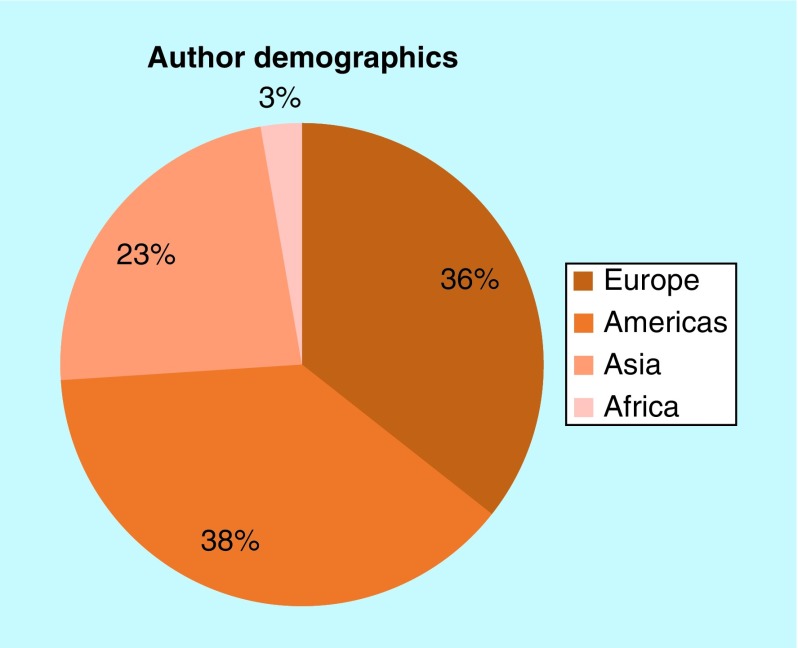
***Future Science OA* author demographics.**

In 2018, our reader demographics were very similar to 2017, which is excellent to see (Figure 2). Our proportion of readers from Asia has increased slightly, with a small decrease in those from the Americas. This is owing to an increase in both the total number of readers and the total number of readers from Asia, and is possibly due, at least in part, to an increase in the amount of articles from researchers based on the continent (Figure 3).

As with our readers, the geographical locations of our authors are relatively steady. Oceania is not represented on the chart this year as it was lower than 1% of authors; however, we have some excellent content in the pipeline, so watch this space.

## Thanks to our contributors

The journal could not be as successful as it is without the time investment made by our contributors. We thank the Editorial Board and Young Ambassador Panel for their continued support of the journal, as well as our authors and peer reviewers for the time they have spent helping us ensure our publications are scientifically sound and relevant. We continue to utilize Publons to reward our reviewers, and to provide discounts on article processing charges as a thank you for their time.

## Looking forward to 2019

With such a good 2018 behind us, we have now begun to look forward to 2019. We have some exciting projects planned already, including a special issue dedicated to early career research, in which we will be publishing insights into maximizing your career, and commentary on the challenges facing early career researchers. If you have a feature you would like to see in 2019, please get in touch.

## References

[1] https://www.scienceopen.com/search#collection/22fcbb40-bc91-44cf-b677-eeaa8d2e3a7f.

[2] The Early Career Research Award 2018 https://www.future-science.com/journals/fso/category/earlycareerresearch/award.

[3] Meet the winner of the 2018 Future Science Early Career Research Award. https://www.future-science-group.com/winner-early-career-research-award.

[4] https://www.chrisxanddrake.com/.

[5] Van Tulleken C, van Tulleken X, Drake J, Wilson H (2018). A note from the ChrisXandDrake Science Award team. *Future Sci. OA*.

[6] Satterfield BA (2017). The future of preventing and treating Nipah virus infection. *Future Sci. OA*.

[7] Ali J, Rafiq QA, Ratcliffe E (2018). Antimicrobial resistance mechanisms and potential synthetic treatments. *Future Sci. OA*.

[8] Andrade JM, Faustino C, Garcia C, Ladeiras D, Reis CP, Rijo P (2018). *Rosmarinus officinalis* L.: an update review of its phytochemistry and biological activity. *Future Sci. OA*.

[9] Holton KF, Cotter EW (2018). Could dietary glutamate be contributing to the symptoms of obsessive-compulsive disorder?. *Future Sci. OA*.

[10] Justo G, Macchiute de Oliveira E, Jurburg C (2018). Functional foods and cancer on Pinterest and PubMed: myths and science. *Future Sci. OA*.

